# Association and mediation analyses among multiple metal exposure, mineralocorticoid levels, and serum ion balance in residents of northwest China

**DOI:** 10.1038/s41598-024-58607-5

**Published:** 2024-04-05

**Authors:** Honglong Zhang, Jun Yan, Guole Nie, Danna Xie, Xingwang Zhu, Jingping Niu, Xun Li

**Affiliations:** 1 The First School of Clinical Medical, Lanzhou University, Lanzhou, 730000 Gansu People’s Republic of China; 2 Department of General Surgery, The First Hospital of Lanzhou University, Lanzhou, 730000 Gansu People’s Republic of China; 3 Key Laboratory of Biotherapy and Regenerative Medicine of Gansu Province, Lanzhou, 730000 Gansu People’s Republic of China; 4 School of Public Health, Institute of Occupational and Environmental Health, Lanzhou University, Lanzhou, 730000 Gansu People’s Republic of China; 5 Department of General Surgery, The First Hospital of Lanzhou University, No.1 Donggang West Road, Chengguan District, Lanzhou, 730030 Gansu China

**Keywords:** Toxic metals, Serum ions, Deoxycorticosterone, Exposure–response relationship, Mediation analysis, Environmental sciences, Environmental social sciences, Medical research, Risk factors

## Abstract

Toxic metals are vital risk factors affecting serum ion balance; however, the effect of their co-exposure on serum ions and the underlying mechanism remain unclear. We assessed the correlations of single metal and mixed metals with serum ion levels, and the mediating effects of mineralocorticoids by investigating toxic metal concentrations in the blood, as well as the levels of representative mineralocorticoids, such as deoxycorticosterone (DOC), and serum ions in 471 participants from the Dongdagou–Xinglong cohort. In the single-exposure model, sodium and chloride levels were positively correlated with arsenic, selenium, cadmium, and lead levels and negatively correlated with zinc levels, whereas potassium and iron levels and the anion gap were positively correlated with zinc levels and negatively correlated with selenium, cadmium and lead levels (all *P* < 0.05). Similar results were obtained in the mixed exposure models considering all metals, and the major contributions of cadmium, lead, arsenic, and selenium were highlighted. Significant dose–response relationships were detected between levels of serum DOC and toxic metals and serum ions. Mediation analysis showed that serum DOC partially mediated the relationship of metals (especially mixed metals) with serum iron and anion gap by 8.3% and 8.6%, respectively. These findings suggest that single and mixed metal exposure interferes with the homeostasis of serum mineralocorticoids, which is also related to altered serum ion levels. Furthermore, serum DOC may remarkably affect toxic metal-related serum ion disturbances, providing clues for further study of health risks associated with these toxic metals.

## Introduction

The acceleration of industrialization development in China has exposed people to various toxic metals through occupational exposure, ambient air, electronic products, and food^1–3^. Toxic metals, such as Pb, Cd, As, Se, Cr, and Cu, have the capability to bioaccumulate in the human body through the food chain and represent a significant threat to human health^4–6^. Although existing studies have reported that toxic metal exposure is related to the occurrence and development of various systemic diseases^7–9^, only a few clinical studies have reported the relationship between serum toxic metal and trace element levels^10,11^. Serum ions, including Na, K, Cl, Ca, Mg, and P, are associated with increased risks of fractures, cardiovascular disease, and chronic kidney disease^12–14^. Currently, serum ion detection serves as a key medical diagnostic technique for guiding clinical diagnosis, treatment, and disease research^15^. Our previous cross-sectional study determined the concentrations of Cd and Pb in whole blood samples and found they could lead to serum ion imbalance in rural residents of China^16^. However, residents may be exposed to various metals, and the pathways through which toxic metals cause serum ion disorders remain unknown. We assume that this may be related to mineralocorticoid levels.

Disrupted mineralocorticoid levels represent a potential pathway through which environmental pollutants may cause adverse health effects on humans^17^. In a Swiss cross-sectional study, a positive relationship was observed between urinary Cd excretion and increased mineralocorticoid metabolite excretion in the general population^18^. Aldosterone and deoxycorticosterone (DOC) are typical mineralocorticoids that play vital roles in maintaining the balance between water and salt metabolism, increasing oxidative stress, and remodeling blood vessels^19–21^. Moreover, DOC serves as the precursor for aldosterone synthesis, and both hormones play a vital role in facilitating Na^+^ reabsorption and K^+^ excretion in renal tubules, which is crucial for regulating water, electrolyte, and blood volume^22^; therefore, they have extensive application in the management of various clinical disorders^23,24^. However, limited studies have focused on the relationship between toxic metal exposure and mineralocorticoid levels in humans, and only a few animal studies have focused on single metal exposure^25^. In addition, to our knowledge, no study has investigated the mediating effects of mineralocorticoids on the relationship between toxic metal exposure and serum ion imbalance in humans.

Humans are simultaneously exposed to numerous metals^26^. However, existing studies have only focused on the relationship between exposure to individual toxic metals and serum ion and mineralocorticoids without considering the synergistic or antagonistic effects of multiple metals^27^. Some researchers have adopted traditional regression models to examine the correlation between exposure to multiple metals and serum ion and mineralocorticoids^28^; however, such regression models may overlook the multicollinearity and variance inflation among metals, leading to inaccurate estimation of the actual impact of metal mixtures^29,30^. Therefore, we applied the weighted quantile sum (WQS)^31^, quantile-based g computation (qgcomp)^32^, and Bayesian kernel machine regression (BKMR) models^33^ in our mixture modeling approach to estimate the combined effects of metal mixtures on health and to evaluate the corresponding contributions of individual metals^34^. Collectively, the analysis of the impact of toxic metals on human health using a multi-exposure model provides vital theoretical support for improving the health of local residents.

In this context, we hypothesize that serum DOC mediates the relationship between metal mixtures and serum ion imbalance. Therefore, the rural residents of Dongdagou–Xinglong (DDGXL) cohort were selected as the research objects in this study, because they were indirectly exposed to toxic metals through long-term consumption of food, vegetables and fruits polluted by heavy metals, which can better understand the impact of long-term low-dose exposure to toxic metals on human health. We measured individual serum ion levels and whole blood toxic metal concentrations at baseline and used serum DOC as a typical biomarker to assess internal mineralocorticoid levels. The purpose of this study was to evaluate the relationship of exposure to eight metals individually and mixed metals with serum ion levels using various mixture modeling methods, and to further investigate the mediating effect of serum DOC in rural residents.

## Materials and methods

### Study areas and population

Two areas in Gansu Province, China, were included in this study. Detailed information about control and polluted areas has been described in our previous study^35^. The residents of the two areas predominantly belong to the Han nationality, and they share similar dietary habits, customs, and culture.

The study participants were selected from a baseline survey of the DDGXL cohort, a prospective cohort aimed to explore the relationship between exposure to toxic metals and associated risks to human health. Long-term local residents who met specific inclusion and exclusion criteria were invited to join the cohort through face-to-face interviews^35^. After obtaining written consent from all the participants, our team visited local clinics or hospitals for health examinations and blood sample collection. A total of 471 participants participated in this study (Fig. S1).

### Blood sample collection and toxic metal measurements

Blood samples were collected from each fasting subjects by research nurses at the Gansu Provincial Biotherapy and Regenerative Medicine Key Laboratory. EDTA-anticoagulated blood tubes were used to collect whole blood. After the blood was collected, the blood tubes were gently inverted several times and then packed into 1.5 mL centrifuge tubes for toxic metal detection. Serum sample collection for ions and mineralocorticoid detection was performed with the use of ordinary blood tubes without anticoagulants. All blood samples were numbered and transferred to a − 80 °C refrigerator in the biobank until testing.

All blood samples were thawed and mixed sequentially before testing. Blood samples (0.2 mL) were mixed with 30% H_2_O_2_ and 65% HNO_3_ solution (0.5 mL), shaken, nitrified using a microwave digestion apparatus (MARS-5, CEM, USA), and then passed through an inductively coupled-mass spectrometer (ICP-MS, PerkinElmer Sciex, USA) for the analysis of eight toxic metal, including Al, Cr, Cu, Zn, As, Se, Cd, and Pb. We use laboratory blanks, labeled mixed blood samples, and standard reference materials from Seronorm™ Trace Elements Whole Blood (SERO AS, Norway) for quality control. The calibration curve, limit of detection (LOD), recovery rate, and relative standard deviation (RSD) of eights metals were listed in Table S1. Additionally, the blood toxic metal concentrations below LODs were assigned with the LOD divided by the square root of 2, according to laboratory documentation.

### Serum mineralocorticoid analysis

High-performance liquid chromatography–tandem mass spectrometry (HPLC–MS/MS) were used for serum mineralocorticoid analysis following the analytical method previously reported in our study^36^. (1) Sample preparation: serum samples (0.2 mL) was fully extracted, centrifuged, and the supernatant was transferred to the brown sample bottle to evaporate until dry. The dried extract was re-suspended with 200 μL of 50% acetonitrile, mixed and filtered, and transferred to an injection vial for LC–MS analysis. (2) Chromatographic conditions: Chromatographic separation was carried out on a Phenomenex Kinetex C18 column (100 mm × 2.1 mm, 1.7 μm, California, USA) at 40 °C with a flow rate of 0.3 mL/min and a total run time of 8 min. The mobile phase and gradient elution procedures are shown in Table S2. (3) Mass spectrometry conditions: Mineralocorticoid was detected using multiple reaction monitoring in electrospray ionization positive mode. The ion source parameters were set as follows: temperature 500 °C, voltage 5.5 kV, gas 1 at 55 psi, gas 2 at 45 psi, curtain gas at 25 psi, and collision-activated dissociation gas at 10 psi. The MRM acquisition parameters are shown in Table S3. (4) Methodological verification: the standard curves of the sample showed good linear results, and the detailed linear regression parameters, LOD, recovery rate, and RSD of DOC were shown in Table S1.

### Serum ions analysis

All serum samples were transported to the Laboratory Department on the day of collection, and the concentrations of serum K, Na, Cl, Ca, P, Mg, Fe, and the anion gap (AG) were measured by professional technicians using an automatic chemistry analyzer (Beckman AU5800, Japan), according to strict operating procedures^16^.

### Statistical analysis

The Kolmogorov–Smirnov and Shapiro–Wilk tests were used to detect normality of continuous variables, and toxic metals, mineralocorticoids, and serum ions were logarithmically transformed for subsequent analyses (Table S4). The basic characteristics of the subjects were described using the Student’s *t-*test, Mann–Whitney U test, and Chi-square test.

The relationship between toxic metal exposure, mineralocorticoids, and serum ions was examined by linear regression analyses. Model 1 was crude model, and Model 2 was further adjusted for age, sex, BMI, waist circumference, smoking, and alcohol consumption, which were all selected according to previous literature. In addition, the effects of exposure to multiple toxic metals on mineralocorticoid and serum ions were analyzed using WQS, qgcomp and BKMR models (R-pack “gWQS”, “qgcomp” and “bkmr”). In the WQS model, a weighted index was constructed to estimate the overall impact of all metals on the outcomes, and the weight of a single toxic metal was calculated to determine its contribution to the overall outcome index^37^. The qgcomp model has the ability to assign a weight (either positive or negative) to each individual toxic metal, so we can consider the interaction between eight metals and the outcomes in either direction^38^. The BKMR model can calculate posterior inclusion probability (PIP) to estimate the relative contribution of a single metal to the final result^39,40^. Together, the three models were used to estimate the combined effects of exposure to numerous environmental pollutants on human health^34,41^.

Finally, mediation models were used to estimate the mediating effects of mineralocorticoids on the correlations of metals with serum ions. Specifically, mediation analysis was used to test whether the independent variable X (toxic metals) had an effect on the dependent variable Y (serum ions) by influencing the intermediary variable M (mineralocorticoids). The results of the mediation analysis enable us to obtain several important indicators, including direct effect (DE), which represents the effect of toxic metals on serum ions without mineralocorticoids; indirect effect (IE), which represents the effect of toxic metals on serum ions through mineralocorticoids; total effect (TE) = DE + IE; Proportion of mediation = IE/TE^42,43^. Furthermore, to test the moderating effect of mineralocorticoids, interaction terms for predicting serum ion levels were created by multiplying single and mixed toxic metals with mineralocorticoid, and the resulting interaction effects were subsequently elucidated using a simple slope test^44^. SPSS 24.0 and R Studio were used for statistical analyses.

### Ethical approval

The study protocol complied with the principles of the Declaration of Helsinki and was approved by the Ethics Committee of First Hospital of Lanzhou University (LDYYLL2015-0027 and LDYYLL2020-103).

### Consent to participate

All participants have signed written informed consent.

## Results

### Basic characteristics of study population

Table 1 summarizes the basic characteristics, toxic metal distribution, serum DOC concentrations, and serum ion levels in all the participants. The mean age of the 471 participants (171 males, 36.31%) was 57.43 years, and the mean BMI was 23.63 kg/m^2^. Males had higher average height, weight, age, education level and tobacco, tea, and alcohol consumption rates, whereas females had higher BMI (*P* < 0.05). The distribution of toxic metals in the blood is shown in Table 1. All toxic metals were detectable. The levels of Al, Cr, Zn, As, Se, and Pb did not show significant differences between the sexes, and blood Cd was considerably higher in males than in females, whereas blood Cu showed the opposite trend. Serum DOC, K, and Fe levels were higher in males, whereas serum P levels were higher in females.

**Table 1:** Socio-demographic characteristics, metal distribution, mineralocorticoid levels, and serum ion concentrations in the subjects.

Variable	All participants (N = 471)	Gender		*P* value
		Male (n = 171)	Female (n = 300)	
Socio-demographic characteristics				
Height (cm)	161.73 ± 8.06	168.99 ± 6.10	157.58 ± 5.80	< 0.001**
Weight (kg)	61.86 ± 9.97	65.95 ± 10.19	59.53 ± 9.07	< 0.001**
BMI (kg/m^2^)	23.63 ± 3.24	23.04 ± 2.94	23.96 ± 3.36	0.003**
Waist circumference (cm)	84.33 ± 9.13	84.98 ± 9.51	83.96 ± 8.91	0.245
Age (years)	57.43 ± 7.15	58.61 ± 7.06	56.76 ± 7.13	0.007**
Education level				< 0.001**
Primary school	289 (61.4)	68 (39.8)	221 (73.7)	
Middle school	129 (27.4)	59 (34.5)	70 (23.3)	
High school	53 (11.3)	44 (25.7)	9 (3.0)	
Family income/person/year (¥)				0.058
< 3000	177 (37.6)	105 (35.0)	72 (42.1)	
3000–10,000	218 (46.3)	138 (46.0)	80 (46.8)	
> 10,000	76 (16.1)	57 (19.0)	19 (11.1)	
Cigarette smoking				< 0.001**
Yes	148 (31.4)	81 (47.4)	67 (22.3)	
No	323 (68.6)	90 (52.6)	233 (77.7)	
Tea drinking				< 0.001**
Yes	199 (42.3)	92 (53.8)	107 (35.7)	
No	272 (57.7)	79 (46.2)	193 (64.3)	
Alcohol consumption				0.011*
Yes	45 (9.6)	24 (14.0)	21 (7.0)	
No	426 (90.4)	147 (86.0)	279 (93.0)	
Metals distribution				
BAl (ng/mL)	174.09 (18.01–489.37)	146.18 (10.18–458.38)	196.47 (28.89–514.93)	0.306
BCr (ng/mL)	123.53 (76.74–138.20)	123.42 (90.43–139.50)	123.61 (72.38–137.90)	0.665
BCu (ng/mL)	831.92 (726.96–924.84)	805.22 (705.59–903.71)	841.78 (736.07–938.34)	0.023*
BZn (ng/mL)	5586.77 (4810.07–6368.35)	5694.29 (5017.12–6483.66)	5549.28 (4718.97–6236.61)	0.067
BAs (ng/mL)	8.47 (3.19–10.77)	8.55 (3.76–10.98)	8.33 (3.12–10.73)	0.645
BSe (ng/mL)	130.93 (94.42–195.65)	133.73 (95.66–190.43)	130.62 (93.70–198.64)	0.991
BCd (ng/mL)	0.90 (0.08–4.50)	2.24 (0.08–5.86)	0.52 (0.08–3.43)	< 0.001**
BPb (ng/mL)	22.25 (12.42–38.34)	24.18 (13.88–41.79)	21.23 (11.28–35.87)	0.061
Mineralocorticoid level				
DOC (ng/mL)	0.58 (0.56–0.60)	0.59 (0.57–0.61)	0.57 (0.56–0.59)	< 0.001**
Serum ions concentrations				
K (mmol/L)	4.35 (4.10–4.64)	4.47 (4.16–4.80)	4.28 (4.07–4.50)	< 0.001**
Na (mmol/L)	143.00 (141.00–144.00)	143.00 (142.00–144.00)	143.00 (141.00–144.00)	0.437
Cl (mmol/L)	103.00 (101.00–105.00)	103.00 (101.00–105.00)	103.00 (101.00–105.00)	0.364
Ca (mmol/L)	2.34 (2.28–2.40)	2.33 (2.28–2.40)	2.35 (2.28–2.40)	0.073
P (mmol/L)	1.10 (0.98–1.23)	0.97 (0.89–1.08)	1.16 (1.07–1.26)	< 0.001**
Mg (mmol/L)	0.88 (0.84–0.92)	0.89 (0.85–0.92)	0.87 (0.83–0.91)	0.054
Fe (µmol/L)	20.38 (16.24–25.57)	22.70 (18.14–28.81)	18.77 (14.96–23.81)	< 0.001**
AG (mmol/L)	18.00 (16.00–19.00)	18.00 (16.00–19.00)	18.00 (16.00–19.00)	0.481

*BMI* body mass index, *BAl* aluminum in blood, *BCr* chromium in blood, *BCu* cuprum in blood, *BZn* zinc in blood, *BAs* arsenic in blood, *BSe* selenium in blood, *BCd* cadmium in blood, *BPb* lead in blood, *DOC* deoxycorticosterone, *K* potassium, *Na* sodium, *Cl* chlorine, *Ca* calcium, *P* phosphorus, *Mg* magnesium, *Fe* iron, *AG* anion gap.

*P < 0.05; **P < 0.01.

### Relationships between metals and serum ion

**Figure 1:**
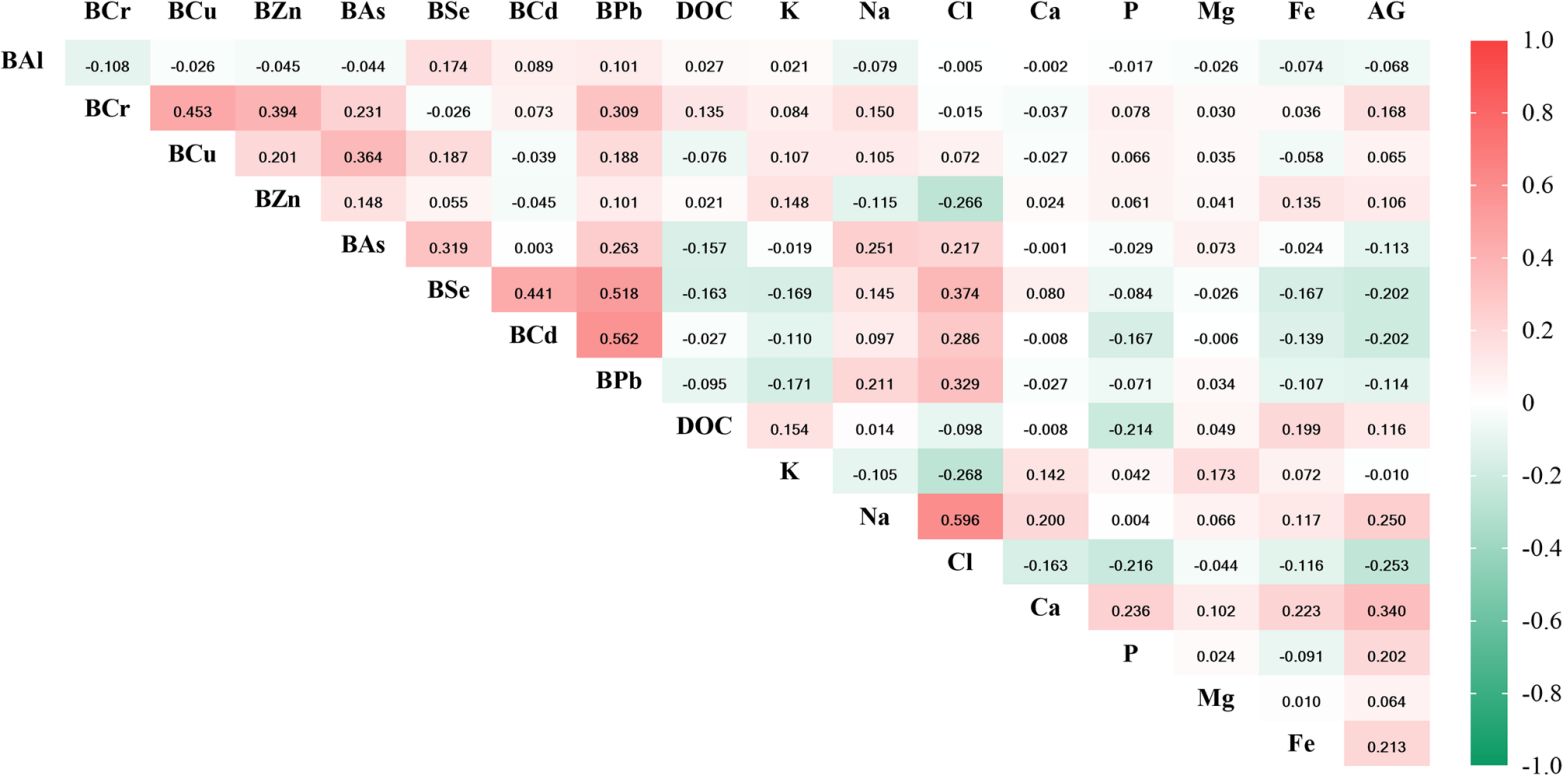
Correlation between blood toxic metals, DOC and serum ion levels analyzed using Spearman’s correlation analysis. *BAl* aluminum in blood, *BCr* chromium in blood, *BCu* cuprum in blood, *BZ*n zinc in blood, *BAs* arsenic in blood, *BSe* selenium in blood, *BCd* cadmium in blood, *BPb* lead in blood, *DOC* deoxycorticosterone, *K* potassium, *Na* sodium, *Cl* chlorine, *Ca* calcium, *P* phosphorus, *Mg* magnesium, *Fe* iron, *AG* anion gap. Toxic metals in blood and serum DOC and ions were logarithmically converted.

Changes in serum ion levels were significantly correlated with toxic metal concentrations (Fig. 1). As was positively correlated with Na and Cl levels but negatively correlated with AG levels (*P* < 0.05). Se, Cd, and Pb were positively correlated with Na and Cl levels but negatively correlated with K, Fe, and AG levels (*P* < 0.05). In addition, significant positive correlations were observed among various toxic metals, and positive and negative correlations were observed among serum ions (*P* < 0.05).

**Figure 2:**
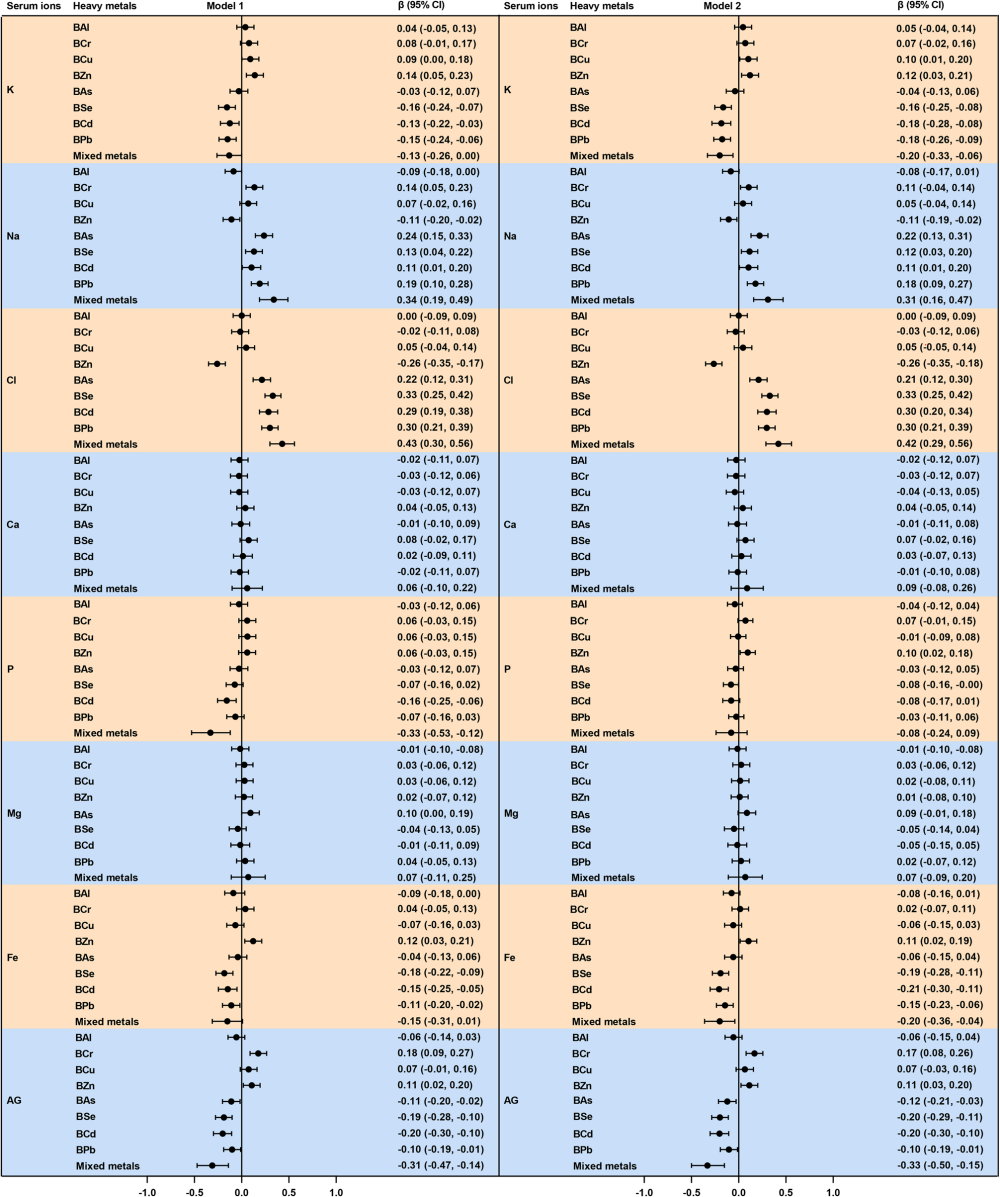
Forest plots showing significant associations of serum ions with single and mixed metal levels in multiple linear regression analysis. Model 1 had no adjustment; Model 2 was adjusted for age, gender, BMI, waist circumference, cigarette smoking and alcohol consumption. *BMI* body mass index, *BAl* aluminum in blood, *BCr* chromium in blood, *BCu* cuprum in blood, *BZn* zinc in blood, *BAs* arsenic in blood, *BSe* selenium in blood, *BCd* cadmium in blood, *BPb* lead in blood, *K* potassium, *Na* sodium, *Cl* chlorine, *Ca* calcium, *P* phosphorus, *Mg* magnesium, *Fe* iron, *AG* anion gap, *CI* confidence interval. Toxic metals in blood and serum ions were logarithmically converted.

Therefore, the relationship of metals to serum ions was analyzed using multiple linear regression models (Fig. 2). Model 1 showed that metals had the greatest effects on serum K, Na, Cl, Fe, and AG. Na and Cl were positively correlated with As, Se, Cd, and Pb levels and K, Fe, and AG were negatively correlated with Se, Cd, and Pb levels (*P* < 0.05). These results remained consistent even after adjusting for multiple confounders in Model 2 (Fig. 2).

### Mixed-exposure analyses

**Figure 3:**
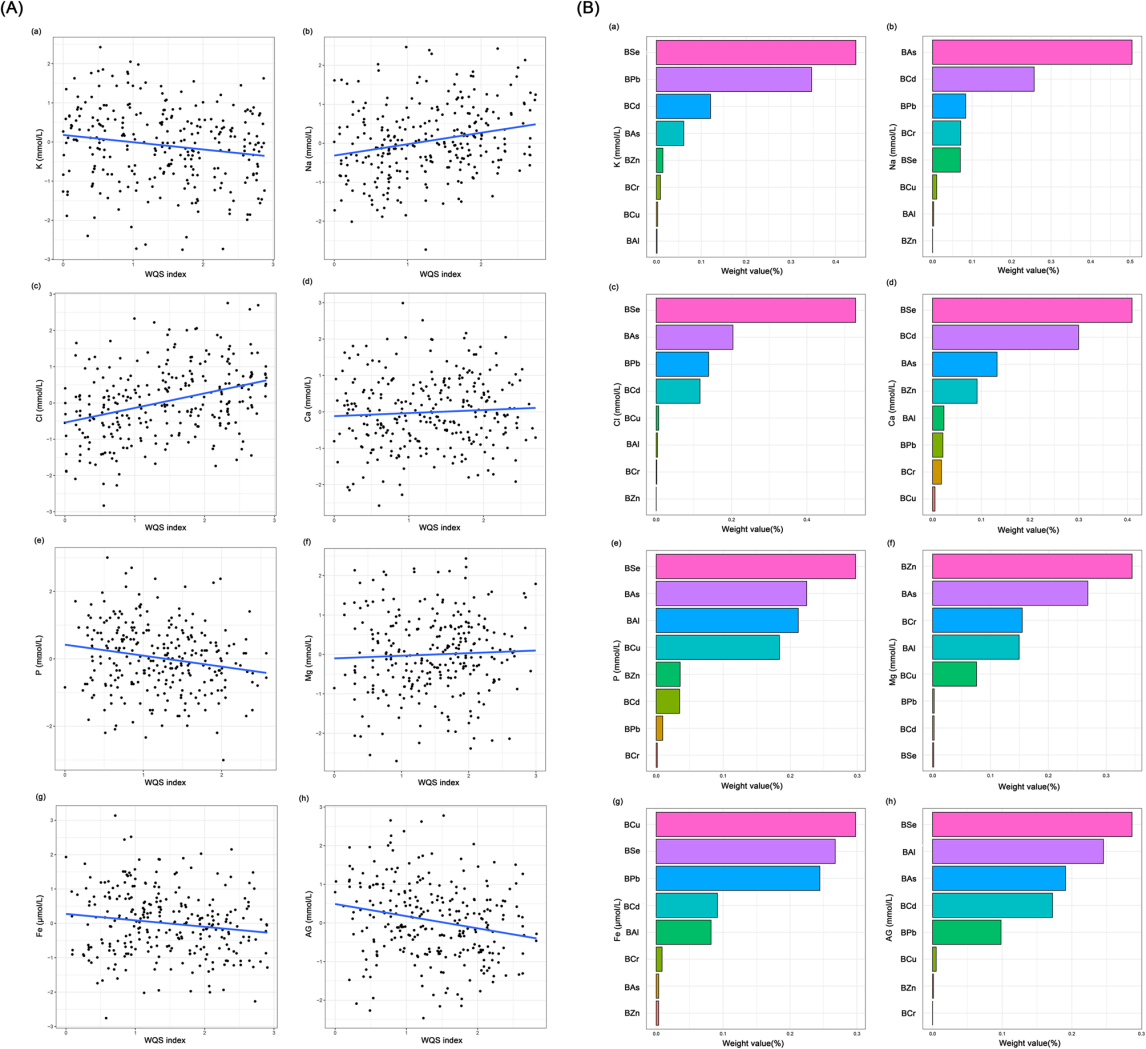
Effects of mixed-exposure to multiple metals on serum ions were evaluated using the WQS model. (**A**) Relationship between WQS index and serum ions. (**B**) Weighted values of toxic metals for serum ions. The model was adjusted for age, gender, BMI, waist circumference, cigarette smoking and alcohol consumption. *BAl* aluminum in blood, *BCr* chromium in blood, *BCu* cuprum in blood, *BZn* zinc in blood, *BAs* arsenic in blood, *BSe* selenium in blood, *BCd* cadmium in blood, *BPb* lead in blood, *K* potassium, *Na* sodium, *Cl* chlorine, *Ca* calcium, *P* phosphorus, *Mg* magnesium, *Fe* iron, *AG* anion gap. Toxic metals in blood and serum ions were logarithmically converted.

In WQS model, the WQS index of mixed metals was positively correlated with Na and Cl levels and negatively correlated with K, Fe, and AG levels, whereas no significant correlation was found with Ca, P, and Mg levels (Fig. 3A). Moreover, the serum K, Na, Cl, Fe, and AG changed levels by − 0.20 (95% Cl − 0.33, − 0.06), 0.31 (95% Cl 0.16, 0.47), 0.42 (95% Cl 0.29, 0.56), − 0.20 (95% Cl − 0.36, − 0.04) and − 0.33 (95% Cl − 0.50, − 0.15), respectively, was observed for per unit increment of WQS scores after adjusting for any confounding factors (Fig. 2). In addition, in the WQS analysis, the weighting of the effects of the eight metals on serum ions are shown in Fig. 3B, among which Se (44.49%), As (50.48%), Se (52.93%), Cu (29.80%), and Se (28.65%) contributed the greatest to serum K, Na, Cl, Fe, and AG respectively.

Similar to the WQS model, toxic metal mixtures showed positive and negative correlations with serum ions in the qgcomp model (Fig. S2A), whereas Cu (32.00%), As (39.27%), Se (35.60%), Cd (37.05%), and Cd (36.45%) contributed the greatest to serum K, Na, Cl, Fe, and AG, respectively (Fig. S2B). Thereafter, the BKMR model was used to estimate the co-effects of toxic metal mixtures. Fig. S3 shows that serum Fe and AG levels decreased with the increase of the toxic metal mixture, while no significant differences were observed for other metals. The conditional PIPs for serum K, Na, Cl, Fe, and AG of the eight toxic metals were all higher than 0.4, indicating that they all contributed to this correlation (Table S5).

### Relationships between toxic metals and DOC levels

**Figure 4:**
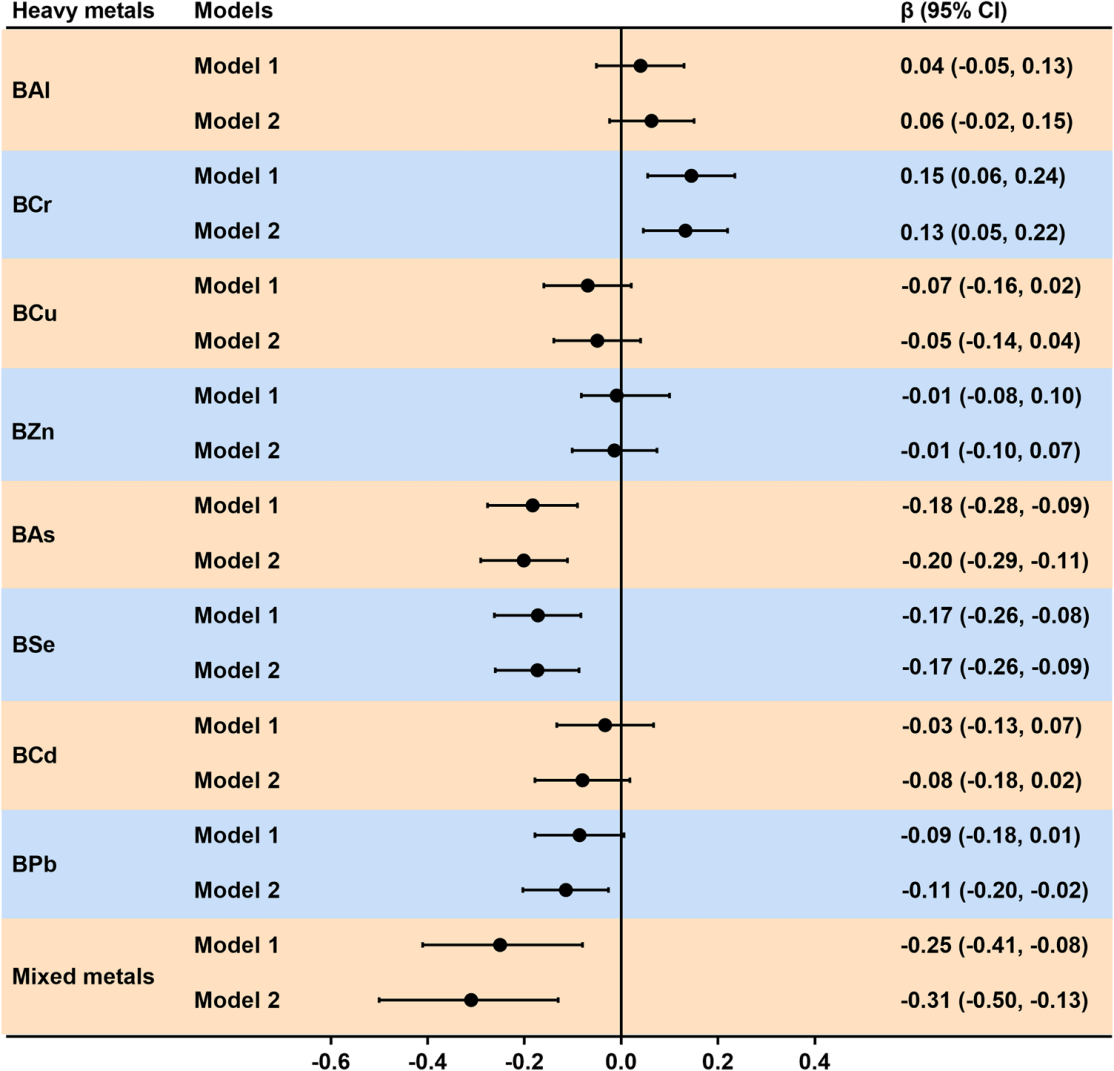
Forest plots showing significant associations of DOC with single and mixed metal levels in multiple linear regression analysis. Model 1 had no adjustment; Model 2 was adjusted for age, gender, BMI, waist circumference, cigarette smoking and alcohol consumption. *BMI* body mass index, *DOC* deoxycorticosterone, *BAl* aluminum in blood, *BCr* chromium in blood, *BCu* cuprum in blood, *BZn* zinc in blood, *BAs* arsenic in blood, *BSe* selenium in blood, *BCd* cadmium in blood, *BPb* lead in blood, *CI* confidence interval. Toxic metals in blood and serum deoxycorticosterone were logarithmically converted.

Serum DOC was negatively correlated with the levels of As, Se, and Pb in blood (*r* =  − 0.157; *r* =  − 0.163; *r *=  − 0.095, respectively) and positively correlated with blood Cr levels (*r* = 0.135) (Fig. 1). Figure 4 shows the positive and negative correlations of Cr, As, Se, and Pb levels in blood with serum DOC levels based on multiple linear regression. We found that the results did not materially change after adjusting for confounding factors in the sensitivity analysis. In the covariate adjusted models, serum DOC was positively correlated with Cr, and was negatively correlated with As, Se and Pb.

**Figure 5:**
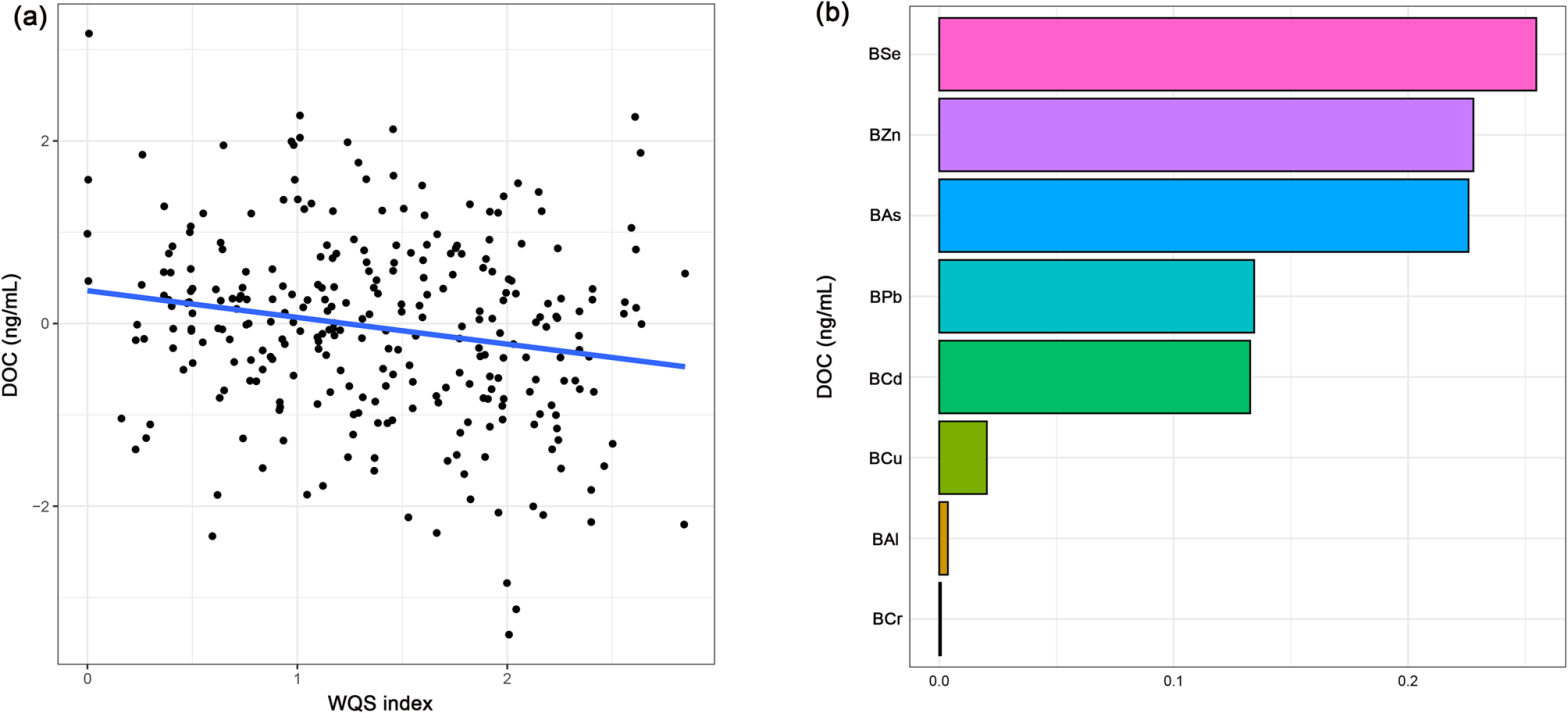
Effects of mixed-exposure to multiple metals on DOC were evaluated using the WQS model. (**A**) Relationship between WQS index and serum DOC. (**B**) Weighted values of toxic metals for serum DOC. The model was adjusted for age, gender, BMI, waist circumference, cigarette smoking and alcohol consumption. *BAl* aluminum in blood, *BCr* chromium in blood, *BCu* cuprum in blood, *BZn* zinc in blood, *BAs* arsenic in blood, *BSe* selenium in blood, *BCd* cadmium in blood, *BPb* lead in blood, *DOC* deoxycorticosterone, *CI* confidence interval. Toxic metals in blood and serum DOC were logarithmically converted.

Evaluation results of WQS regression regarding the correlation between metal mixtures and serum DOC are shown in Fig. 4. The WQS regression index was negatively correlated with serum DOC level (*P* < 0.001), with the top three contributors being Se, Zn, and As (25.47%, 22.79%, and 22.58%, respectively) (Fig. 5). The results of the qgcomp model showed that As (40.61%), Pb (31.95%), and Se (10.95%) contributed the greatest to the serum DOC levels (Fig. S4). Moreover, the BKMR model showed a negative relationship between toxic metal mixtures and serum DOC levels (Fig. S5). We also find that Cr, As, and Pb contributed the greatest to this association because their PIP values were all higher than 0.9 (Table S6).

### Relationship between serum DOC and ion levels

**Figure 6:**
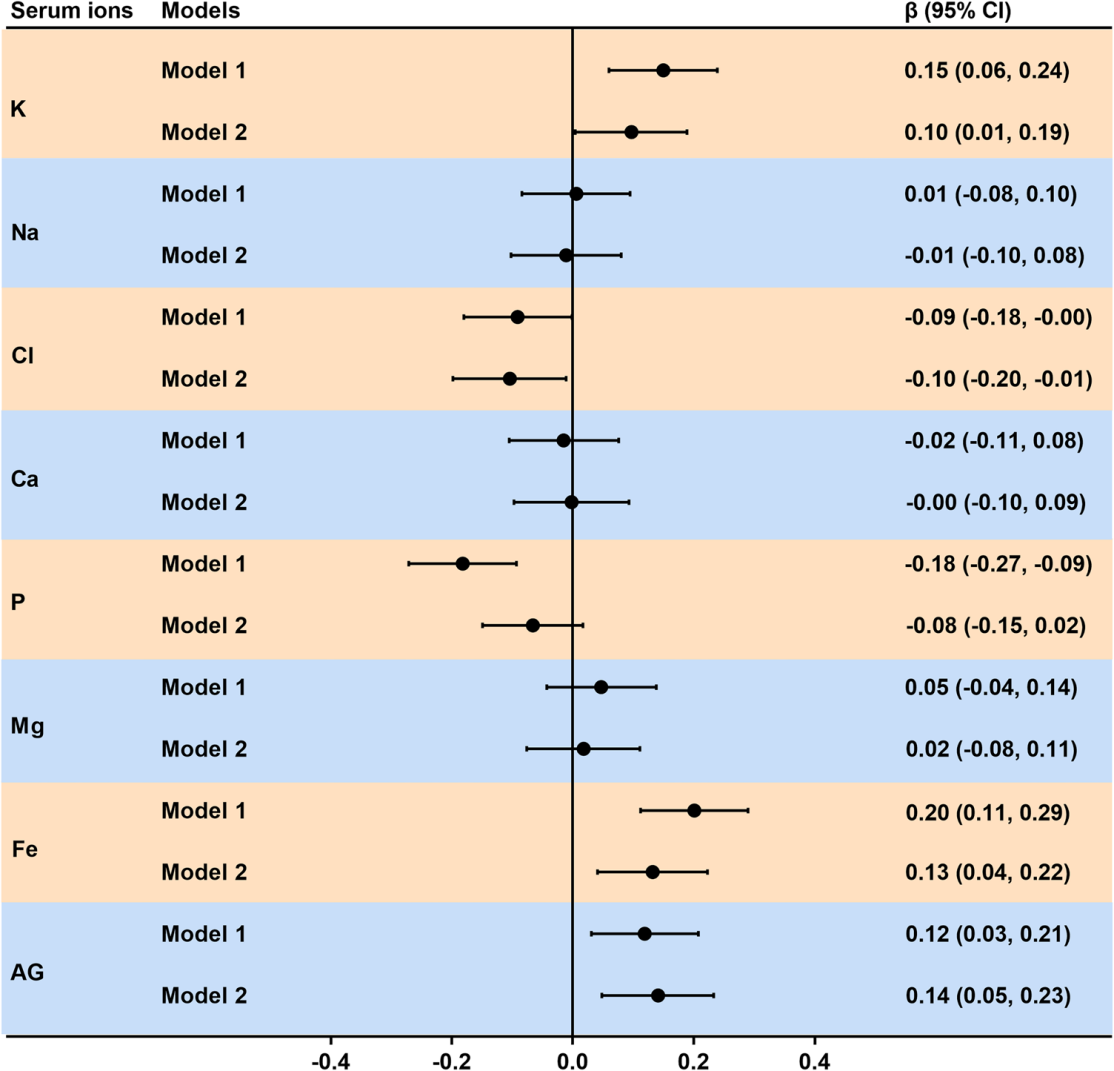
Forest plots showing significant associations of serum ions with DOC levels in multiple linear regression analysis. Model 1 had no adjustment; Model 2 was adjusted for age, gender, BMI, waist circumference, cigarette smoking and alcohol consumption. *BMI* body mass index, *DOC* deoxycorticosterone, *K* potassium, *Na* sodium, *Cl* chlorine, *Ca* calcium, *P* phosphorus, *Mg* magnesium, *Fe* iron, *AG* anion gap, *CI* confidence interval. Serum DOC and ions were logarithmically converted.

Correlation between serum DOC concentration and each ion level was shown in Figs. 1 and 6. A doubling of serum DOC was correlated with 0.15 (95% CI 0.06, 0.24), 0.20 (95% CI 0.11, 0.29), and 0.12 (95% CI 0.03, 0.21) increase in serum K, Fe, and AG concentrations, respectively. Moreover, the increase in serum DOC concentration may be related to the decrease in serum Cl and P levels; however, no correlation was found between serum DOC concentration and the levels of Na, Ca, and Mg. In addition, after adjusting for different covariates in the sensitivity analysis (Fig. 6), we found that our results were robust.

### The mediating effect of serum DOC

**Figure 7:**
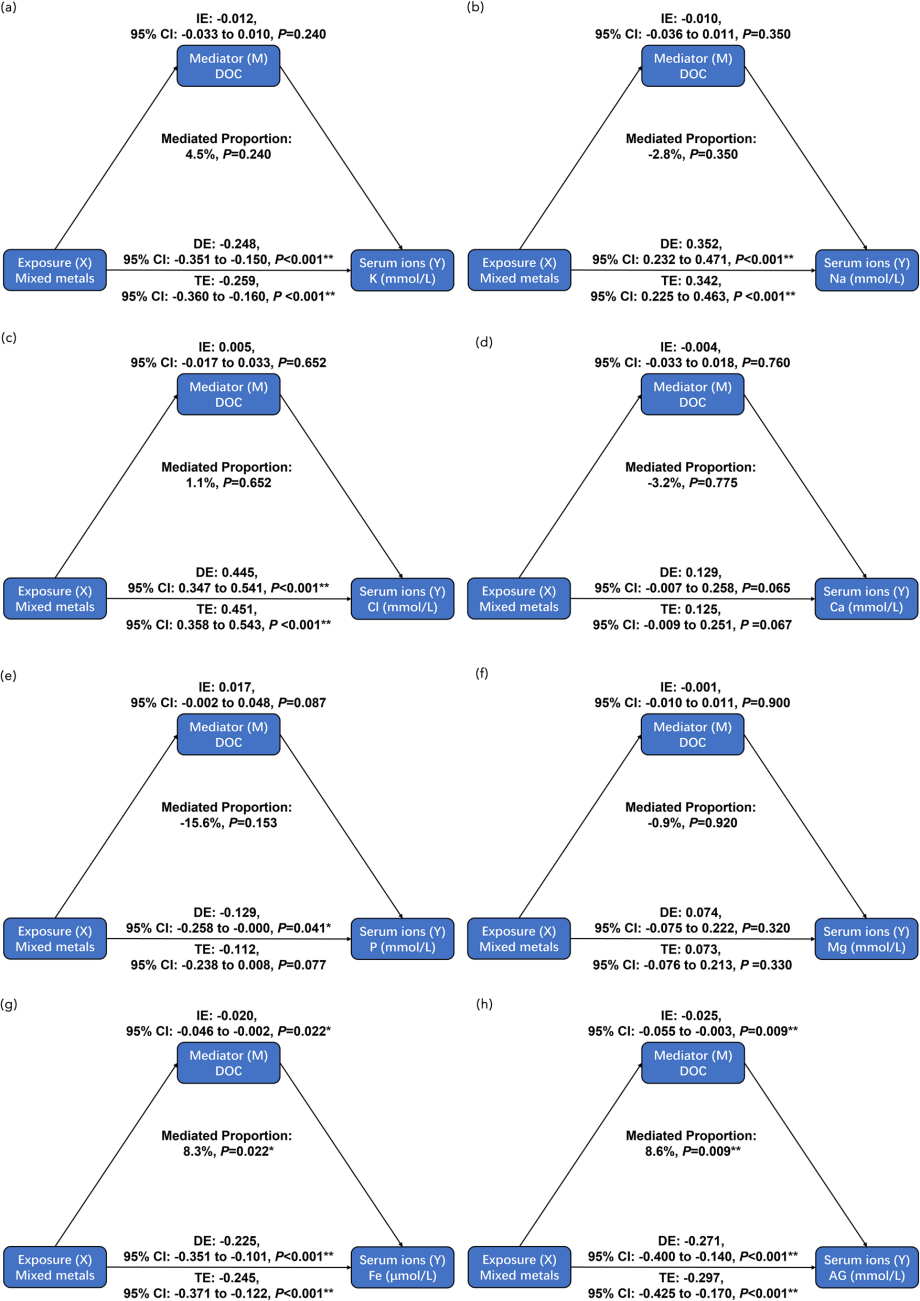
Mediation effect of DOC on associations of mixed metal levels with serum ions. Model was adjusted for age, gender, BMI, waist circumference, cigarette smoking, and alcohol consumption. *BMI* body mass index, *DOC* deoxycorticosterone, *K* potassium, *Na* sodium, *Cl* chlorine, *Ca* calcium, *P* phosphorus, *Mg* magnesium, *Fe* iron, *AG* anion gap, *CI* confidence interval, *IE* indirect effect, *DE* direct effect, *TE* total effect. Proportion of mediation = IE/TE. Toxic metals in blood and serum DOC and ions were logarithmically converted. *P < 0.05, **P < 0.01.

Subsequently, we evaluated the role of serum DOC in the relationship between metals and serum ions using cross-product terms in moderation effect analyses. However, no significant interactions were observed between serum DOC and single or mixed metals (Figs. S6, S7). Finally, we performed mediation analyses to test whether the relationship between metals and serum ions was mediated by serum DOC. The results demonstrated that serum DOC concentration had significant mediating effects on the relationships between Se and Pb with serum Fe levels, and the proportions of mediation were 8.9% and 9.2%, respectively (both *P* < 0.05). The mediating proportions of serum DOC were 9.4%, 20.2%, 9.5%, and 14.5% for the correlations of Cr, As, Se, and Pb levels with serum AG levels, respectively (Fig. S8). Additionally, we found that serum DOC mediated the relationship between mixed metals and serum Fe levels with indirect effects (95% CI) of − 0.020 (− 0.046, − 0.002), as well as the relationship between mixed metals and serum AG levels with indirect effects (95% CI) of − 0.025 (0.055, − 0.003). Elevated serum DOC levels significantly mediated 8.3% and 8.6% of the mixed metal-related reduction in serum Fe and AG levels, respectively (Fig. 7).

## Discussion

In this study, three co-exposure models were used for the first time to assess the relationship between toxic metal mixed-exposure and serum ion disorders in Chinese residents and to explore the mediating role of serum DOC. We found that single or co-exposure to toxic metals was correlated with imbalances in serum ion levels and decreased DOC concentrations after adjusting for potential confounding factors. Furthermore, we identified biological serum DOC as a potential mediator in the correlation of metals, especially mixed metals, with serum ions, which made mineralocorticoids as a potential mechanism for the adverse effects of metals on serum ions.

Our findings are critical for the development of public health. The participants in this study were not occupationally exposed workers, but long-term exposure to toxic metals has been shown to promote disease development^45^. Therefore, we assessed eight metals, mainly from mining and smelting areas, whose concentrations in local soils, crops, and vegetables far exceeded the standard^46,47^. The distribution of metals in blood has been described in previously published studies but it varies. Our results show that the blood toxic metal concentrations of residents in the control area are consistent with those of the general population worldwide^48–52^, whereas the concentrations of Cd, Pb, As, and Se in blood of residents in polluted areas are higher than those of the general population in the control areas and other studies. This discrepancies may be related to environmental pollution and lifestyle factors. Therefore, evaluating the correlation between different levels of toxic metals in blood and serum ions in different populations is crucial.

Our previous study found Cd and Pb co-exposure was positively correlated with dose–dependent increase in serum Na and Cl levels and caused reductions in serum K, P, Fe, and AG levels^16^. Evidence from population studies and animal experiments also supports our findings and has detailed the harmful effects of Cd and Pb exposure, which can cause serum ion imbalances^53–56^. On this basis, we included eight metals: Al, Cr, Cu, Zn, As, Se, Cd, and Pb, some of which are globally known toxic metals. An animal study in poultry demonstrated that exposure to As and/or Cu caused ion profile disorders, including increased Mg, Cr, Se, and Fe, and reduced Ca, Ni, and molybdenum (Mo), whereas elevated Na and K levels were observed only in the As exposure group^57^. Excessive ingestion of chromium chloride (CrCl_3_) could also disrupt the absorption and balance of trace elements in the serum in animal experiments^58,59^. Consistent with the above studies, we found that As, Se, Cd, and Pb were positively correlated with serum Na and Cl levels and negatively associated with serum K, Fe, and AG levels, whereas Zn level was negatively associated with serum Na and Cl levels and positively correlated with serum K, Fe, and AG levels. These results indicate the interaction between toxic metals and serum ion homeostasis.

It is noteworthy that people are often simultaneously exposed to numerous metals in the environment, and their synergistic or antagonistic effects on the disease has been mentioned in previous studies^60,61^. Therefore, the interactions between metal mixtures may also have a greater or weaker impact on the serum ion spectrum. A toxicological study reported that high doses of toxic metal mixtures cause electrolyte balance disturbances in rats^62^. However, animal studies have focused on two or more toxic metals^63,64^, and it is difficult to simulate human exposure to nearly ten toxic metals in a real environment. Thus, we performed a common exposure analysis using the WQS, qgcomp, and BKMR models. The results consistently showed that the eight metal mixtures had significant combined effects on serum K, Na, Cl, Fe, and AG levels. In fact, the WQS model forces the effects of each exposure in the mixture in the same direction^65^, whereas the qgcomp model allows the weights to move in different directions^66^, and the BKMR model overcomes the disadvantages of traditional methods, which may be limited by multicollinearity and model selection errors^41^. Therefore, the combination of these three approaches appears to more accurately reflect the combined health effects of complex exposures and is valuable in identifying important factors. Our findings indicate that serum Na and Cl levels increased as mixed metal concentrations increased, whereas serum K, Fe, and AG levels decreased. Among these, Cd, Pb, As, and Se have greatest contributions. However, serum ion levels are affected by many factors, such as age, diet, and chronic diseases^67–69^, and our findings need to be confirmed in further studies.

The potential mechanism of toxic metal-induced serum ion imbalance is more concerned by us. Firstly, the interaction between metallic elements is considered to be one of the key mechanisms. Since metallic elements share similar physical and chemical properties, they may induce metal interactions at cellular targets, transporter binding sites, and storage sites through the mechanism of ion mimicry^70^. Secondly, toxic metals may disrupt the redox balance to exert their cytotoxic effects. Many studies have shown that toxic metals can damage the normal physiological functions of liver, kidney and other organs by producing excessive reactive oxygen species (ROS) and reducing antioxidant defense ability, etc.^43,71^. Therefore, the imbalance of oxidative stress system may be the potential cause of toxic metals destroying serum ion homeostasis. This study suggests that mineralocorticoid metabolism plays an important role in the disturbance of serum ion levels induced by toxic metals. Mineralocorticoids are the key drivers of electrolyte balance and are crucial for metabolism, cardiovascular function, and body temperature regulation^72,73^. DOC is a representative mineralocorticoid that is comparable to aldosterone and promotes Na reabsorption and K excretion in the distal renal tubules. It is also used as a clinical substitute for primary adrenocortical hypofunction^24^. Nishiyama et al. found that increased plasma aldosterone was the main factor responsible for water and Na retention in rats treated with toxic metals^74^. To confirm the potential of mineralocorticoids for metal-associated serum ion changes, we evaluated the potential role of serum DOC in population-based studies, which confirmed that serum DOC has a considerable effect on serum K, Cl, Fe, and AG levels and that it partially mediates the correlation of mixed metals with serum Fe and AG levels. The results of this study confirm that the imbalance of mineralocorticoid metabolism may be an important way for toxic metals to impair electrolyte homeostasis, but further animal experiments are needed to verify the mechanism found in this study.

More and more scholars pay attention to the effect of toxic metals on mineralocorticoid metabolism. Our results showed that both single metal (As, Se, and Pb) and mixed metal exposure is negatively correlated with serum DOC concentrations. Consistent toxicological evidence suggests that toxic metals can decrease serum aldosterone concentrations in rats^75^. Additionally, a population-based study indicated that critical environmental toxic metal pollution leads to higher levels of mineralocorticoids and an increased risk of cardiovascular disease^28^. The reason for this inconsistency with our results may be the difference in the study population. The above study subjects were myocardial infarction patients, and their serum electrolyte and aldosterone levels had exceeded the normal reference range, while our study subjects were general normal population. Collectively, the few studies available support the finding that toxic metals regulate mineralocorticoid levels. This is the first cross-sectional study to comprehensively analyze the correlation between toxic metals, mineralocorticoid, and serum ion levels in a population sample, further complements the shortcomings in this area.

The mechanism by which toxic metal exposure leads to mineralocorticoid disorder remains unclear. In recent years, many metals, including Cd, As, Pb, Zn, and Cu, have been considered as environmental endocrine disruptors, which can competitively bind to mineralocorticoid receptors (MR), inhibit MR transactivation, and thus alter the expression of mineralocorticoid responsive genes^76^. Moreover, certain metals can also affect 11β-hydroxysteroid dehydrogenase catalyzed synthesis and prereceptor regulation pathways of corticosteroids to interfere with hormone metabolism and homeostasis^77^. In vitro studies have confirmed that Cd, Pb, and Mn can antagonize MR activity, thus significantly reversing the growth inhibition of hepatocellular carcinoma cells induced by aldosterone^78^. In addition, toxic metals can also play a toxic role by inducing mitochondrial damage, oxidative stress, apoptosis^79,80^. However, the relationship between toxic metals and mineralocorticoid disorder needs further study.

The major advantage of our study is the application of WQS, qgcomp, and BKMR models to analyze the damaging effect of multiple toxic metal exposures on serum ion homeostasis, which is more convincing than the single exposure model. In addition, we used DOC to represent human mineralocorticoid levels and applied a mediated analysis model to explore the potential role of mineralocorticoids in the process of toxic metal-induced serum ion disturbance, which has not been previously mentioned.

However, some limitations need to be further addressed. First, this study can’t conclude a causal relationship between toxic metal exposure and serum ion interference between toxic metal exposure and serum ion disturbance; therefore, further longitudinal analysis of repeated measures of these factors is needed. Second, the concentration of toxic metals in blood may not accurately reflect the level of long-term environmental exposure; however, blood samples are the most common in clinical practice and can be used for the detection of multiple indicators. Moreover, future studies will be conducted to measure toxic metals in blood, urine, and the environment to fully assess the risk of toxic metals in humans. Third, diet, exercise, and lifestyle may have a short-term effect on serum ion levels, but the population in this study had lived in rural areas for a long time, and their lifestyle was simple and stable. Therefore, we adjusted for potential confounding factors in our analysis. Finally, we explored the potential role of DOC through mediation analysis, however, the detailed biological mechanisms need to be explored in future animal experiments to verify the findings of this study.

## Conclusion

**Figure 8:**
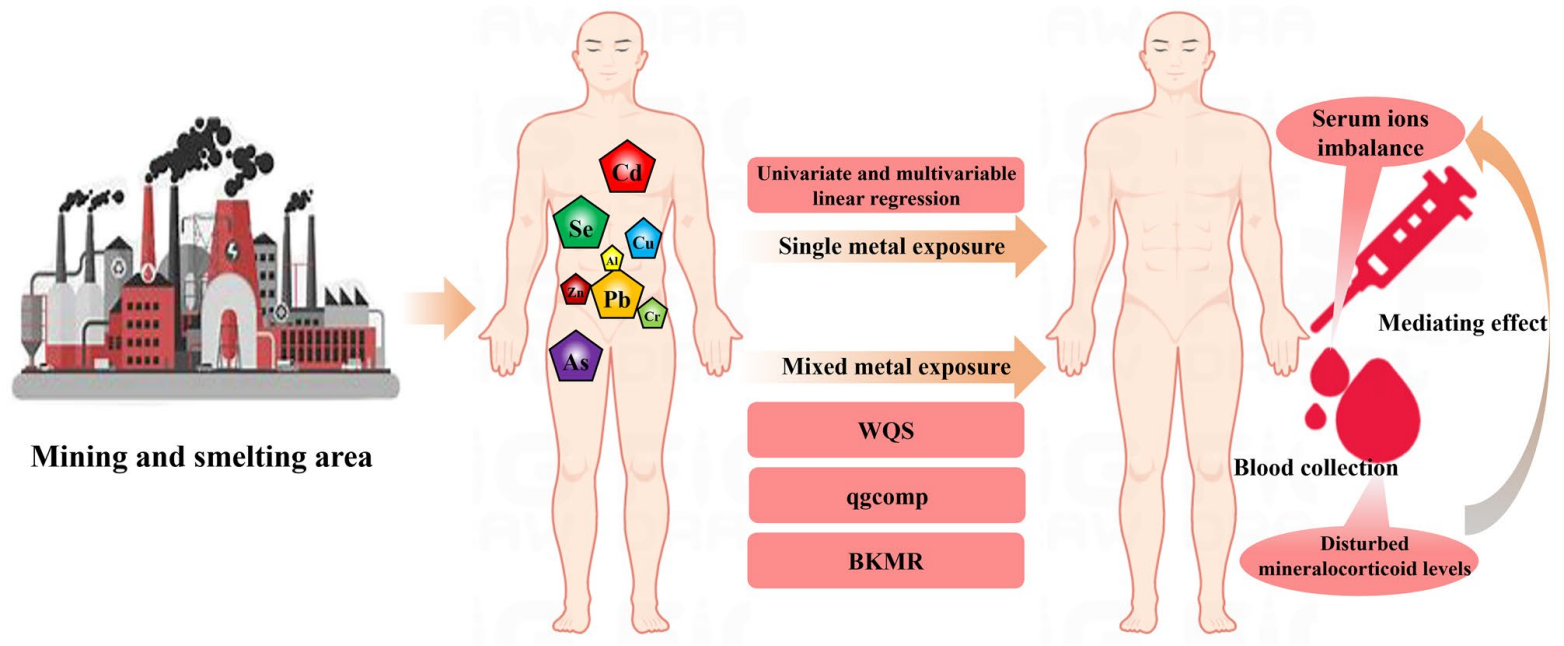
Single and mixed metal exposure interferes with the homeostasis of serum mineralocorticoids, which is also related to altered serum ion levels.

In summary, we found that single and mixed metals were correlated with serum ion imbalances in rural residents of Northwest China, primarily driven by Cd, Pb, As, and Se. Furthermore, significant dose–response correlations of serum DOC with both toxic metal concentrations and serum ions were observed, providing evidence in humans for the first time that serum DOC partially mediates the correlation of toxic metals with serum ion disorders (Fig. 8). Our findings will help create public awareness regarding toxic metal pollution and accordingly help in taking preventive measures to reduce toxic metal exposure. Further studies are required to validate our finding.

### Supplementary Information

Supplementary Information 1.

Supplementary Information 2.

## Data Availability

The datasets generated during and/or analysed during the current study are available from the corresponding author on reasonable request.
