# Supplementary material for: Association and mediation analyses among multiple metal exposure, mineralocorticoid levels, and serum ion balance in residents of northwest China
Source: Sci Rep. 2024 Apr 5;14:8023. doi: 10.1038/s41598-024-58607-5 (PMC10997635; doi:10.1038/s41598-024-58607-5)
Supplement: Supplementary file 2 — Supplementary Information 2. [file 41598_2024_58607_MOESM2_ESM.docx]

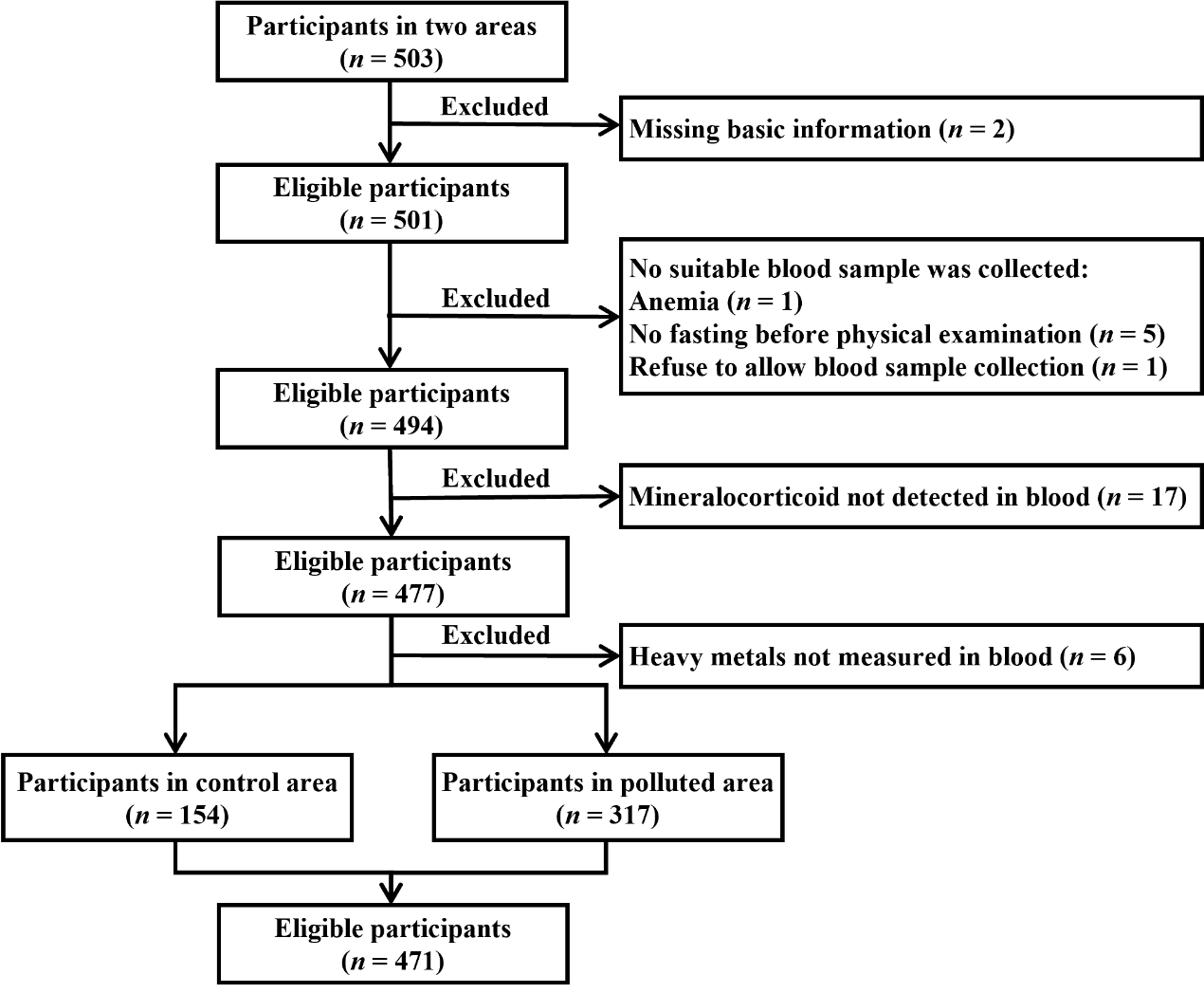


Fig. S1 Flow chart for the participants who were finally included in this study.


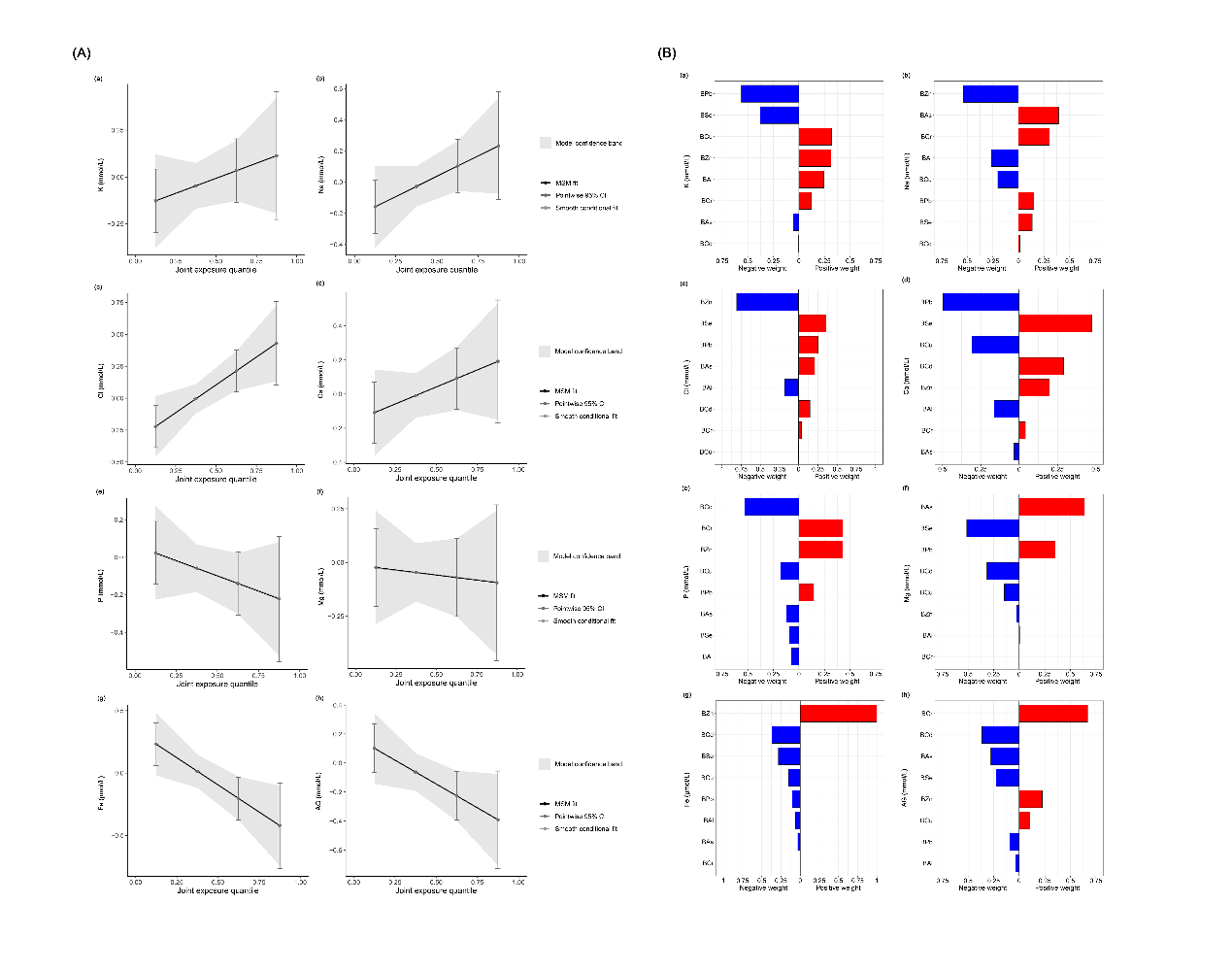


Fig. S2. Effects of co-exposure to multiple toxic metals on serum ions were evaluated using the qgcomp model. (A) The relationship between the qgcomp index and serum ion levels. (B) Estimated risk and weighted values of toxic metals for serum ions by qgcomp models. The model was adjusted for age, gender, BMI, waist circumference, cigarette smoking and alcohol consumption. BAl: Aluminum in blood; BCr: Chromium in blood; BCu: Cuprum in blood; BZn: Zinc in blood; BAs: Arsenic in blood; BSe: Selenium in blood; BCd: Cadmium in blood; BPb: Lead in blood; K: Potassium; Na: Sodium; Cl: Chlorine; Ca: Calcium; P: Phosphorus; Mg: Magnesium; Fe: Iron; AG: Anion gap. Toxic metals in blood and serum ions were logarithmically converted.


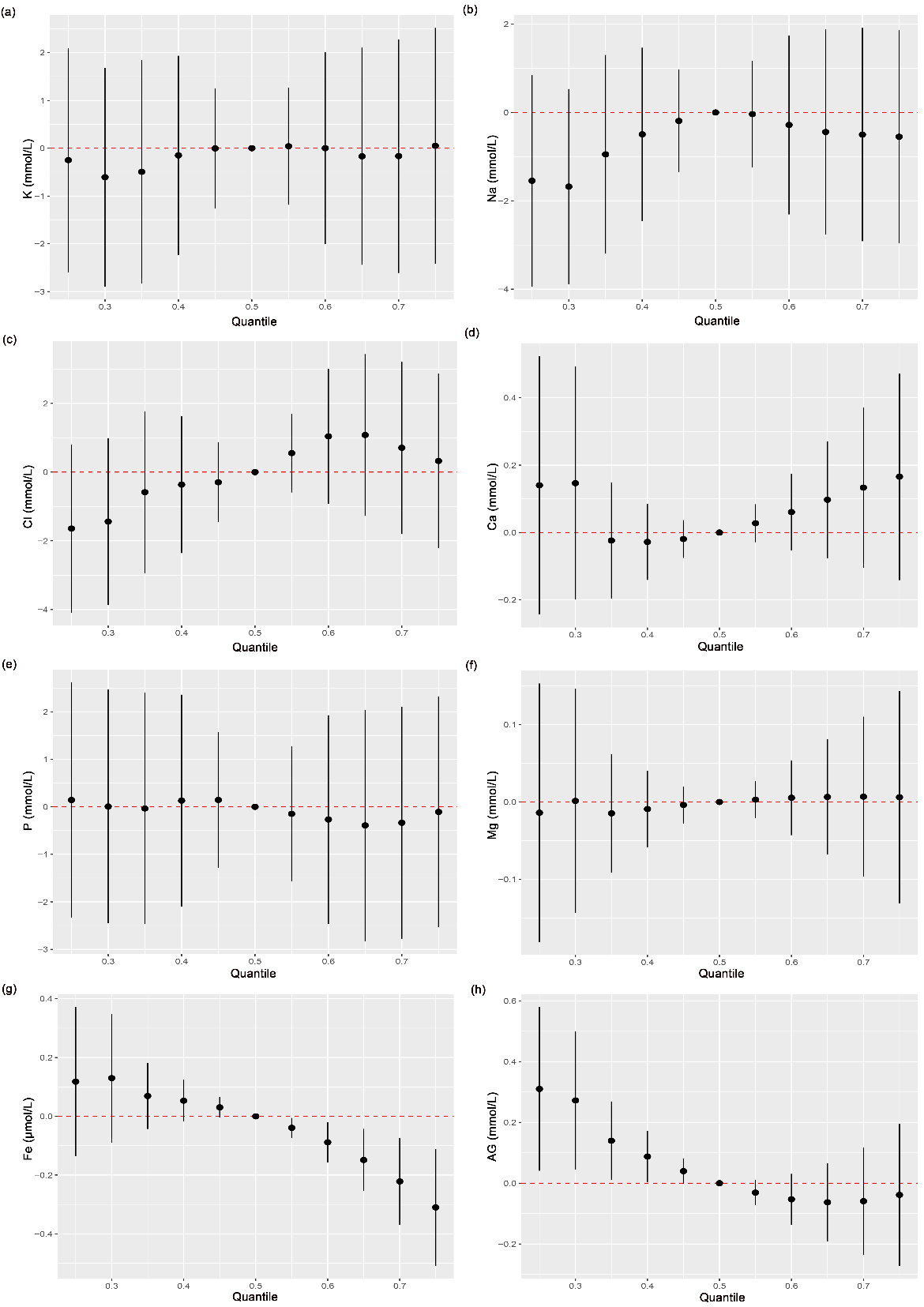


Fig. S3. Effects of co-exposure to multiple toxic metals on serum ions were evaluated using the BRMR model. The model was adjusted for age, gender, BMI, waist circumference, cigarette smoking and alcohol consumption. K: Potassium; Na: Sodium; Cl: Chlorine; Ca: Calcium; P: Phosphorus; Mg: Magnesium; Fe: Iron; AG: Anion gap. Toxic metals in blood and serum ions were logarithmically converted.


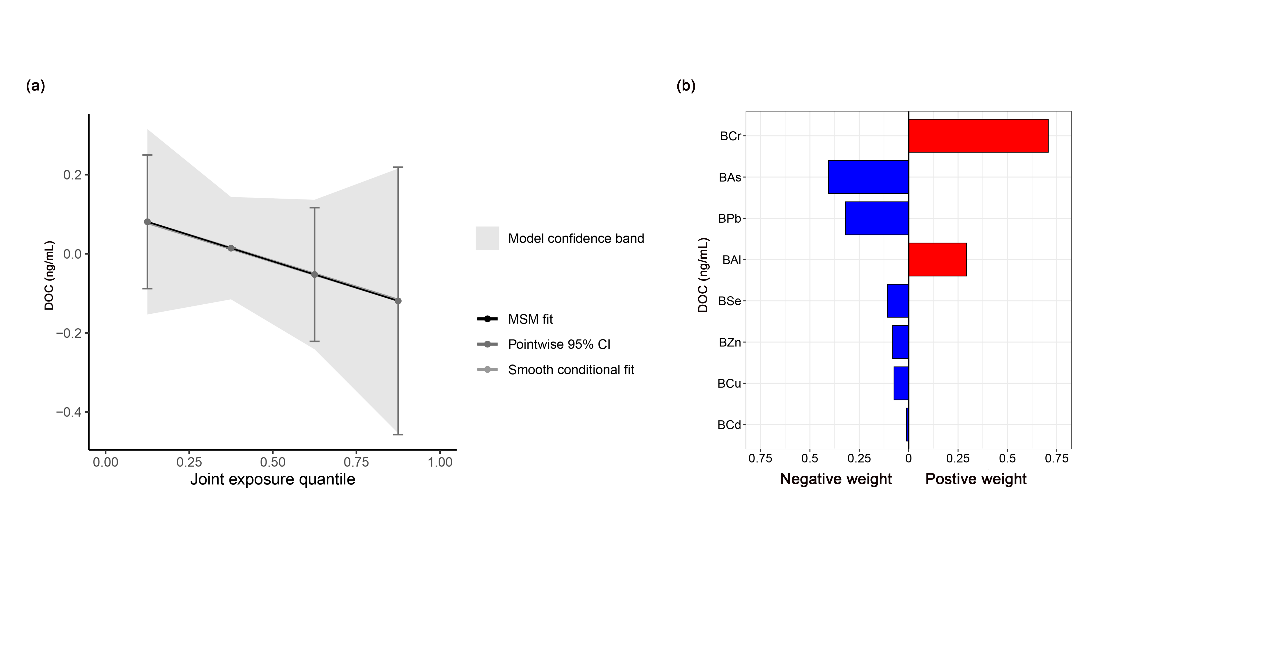


Fig. S4. Effects of co-exposure to multiple toxic metals on DOC were evaluated using the qgcomp model. (A) The relationship between the qgcomp index and serum DOC levels. (B) Estimated risk and weighted values of toxic metals for serum DOC by qgcomp models. The model was adjusted for age, gender, BMI, waist circumference, cigarette smoking and alcohol consumption. BAl: Aluminum in blood; BCr: Chromium in blood; BCu: Cuprum in blood; BZn: Zinc in blood; BAs: Arsenic in blood; BSe: Selenium in blood; BCd: Cadmium in blood; BPb: Lead in blood; DOC: Desoxycorticosterone. Toxic metals in blood and serum DOC were logarithmically converted.


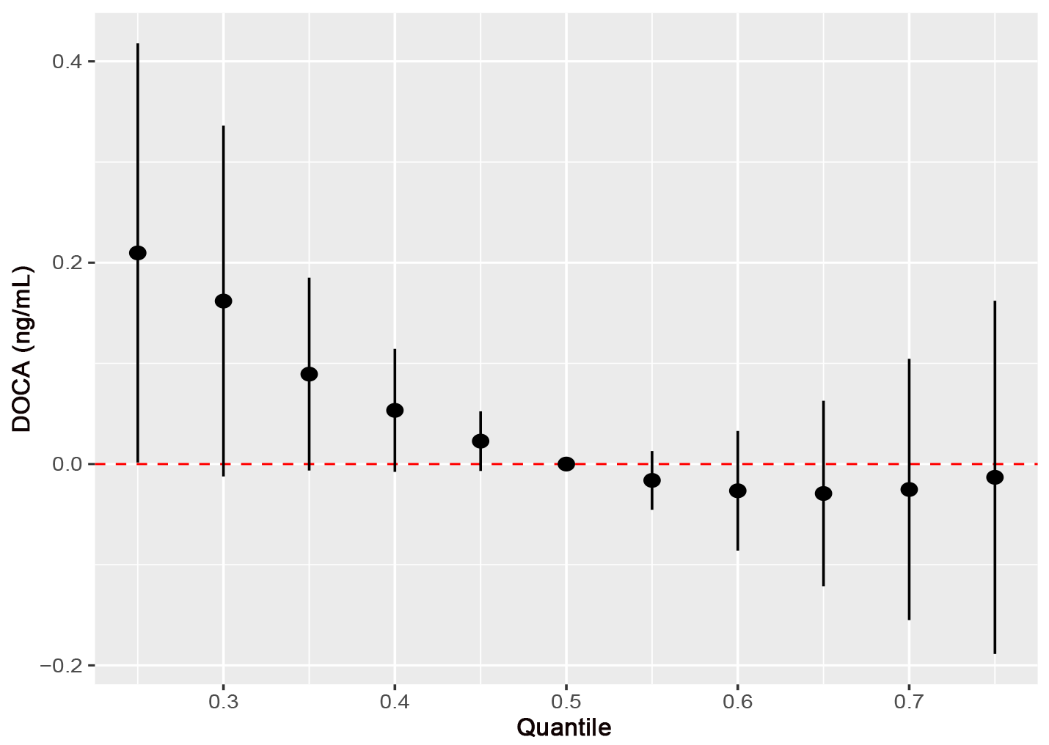


Fig. S5. Effects of co-exposure to multiple toxic metals on DOC were evaluated using the BRMR model. The model was adjusted for age, gender, BMI, waist circumference, cigarette smoking and alcohol consumption. DOC: Desoxycorticosterone. Toxic metals in blood and serum DOC were logarithmically converted.


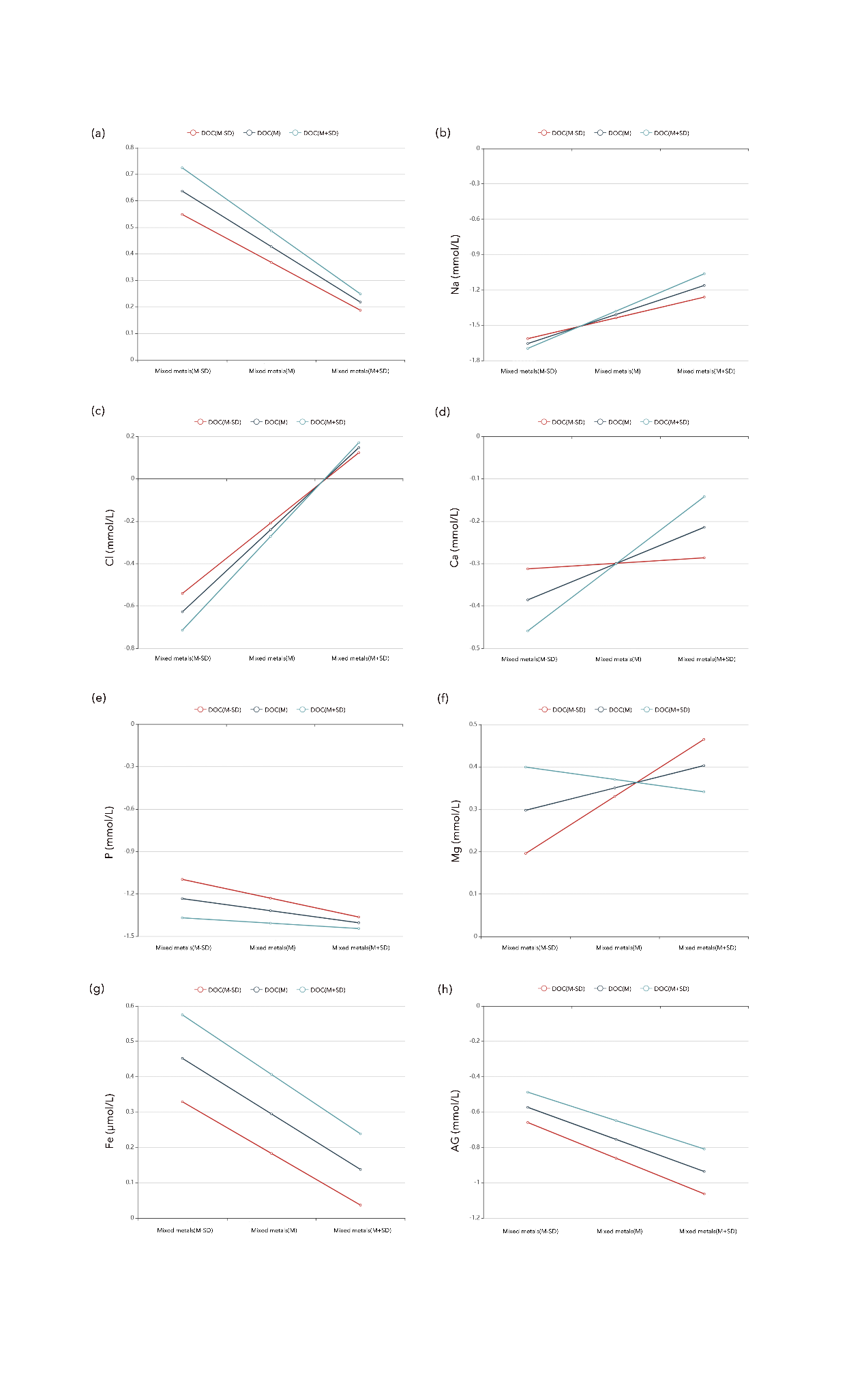


Fig. S6. Moderation effect of DOC on associations of mixed metal levels with serum ions. Model was adjusted for age, gender, BMI, waist circumference, cigarette smoking, and alcohol consumption. DOC: Deoxycorticosterone; K: Potassium; Na: Sodium; Cl: Chlorine; Ca: Calcium; P: Phosphorus; Mg: Magnesium; Fe: Iron; AG: Anion gap; M: mean; SD: standard deviation. Toxic metals in blood and serum DOC and ions were logarithmically converted.


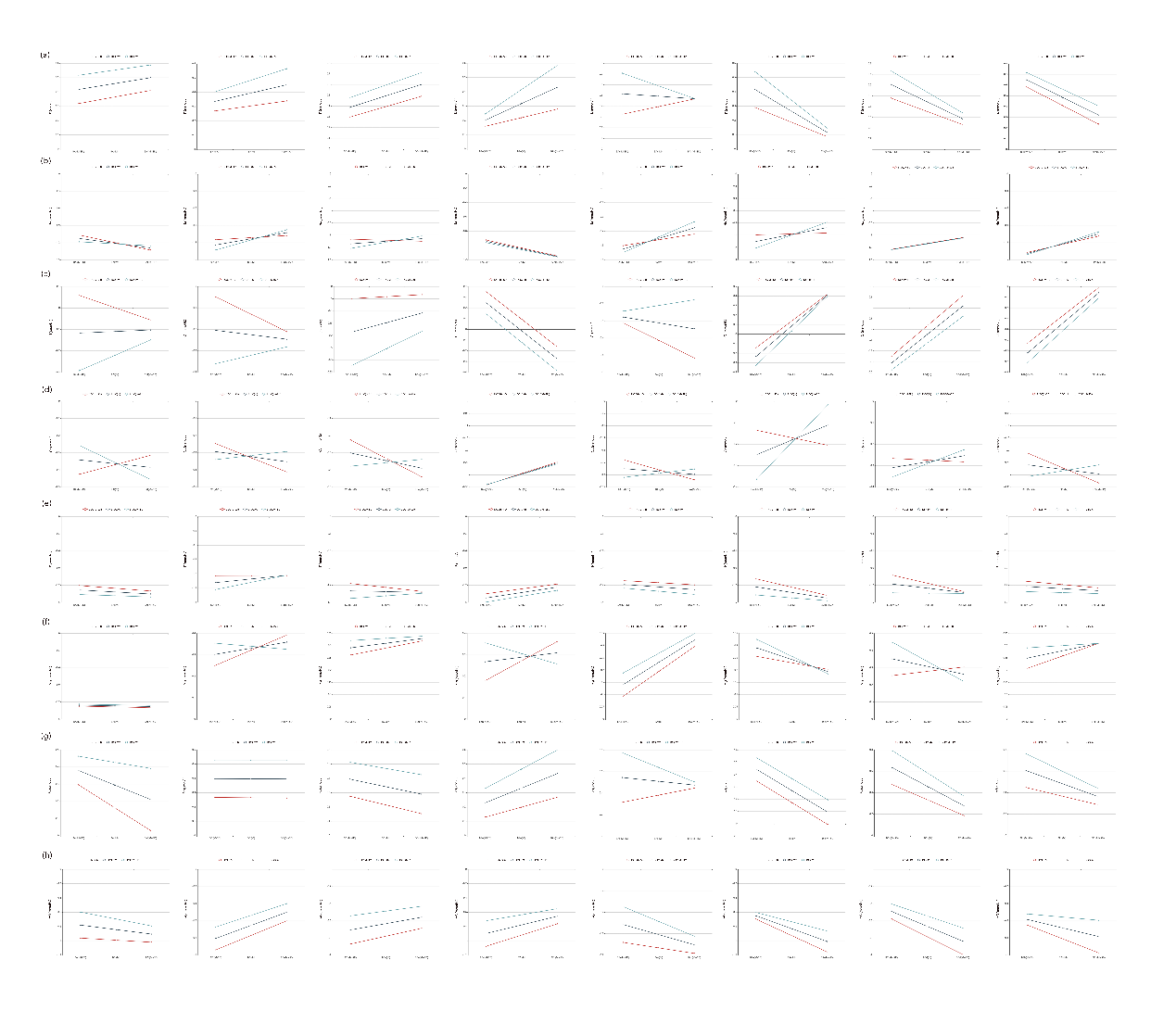


Fig. S7. Moderation effect of DOC on associations of single metal levels with serum ions. Model was adjusted for age, gender, BMI, waist circumference, cigarette smoking, and alcohol consumption. BAl: Aluminum in blood; BCr: Chromium in blood; BCu: Cuprum in blood; BZn: Zinc in blood; BAs: Arsenic in blood; BSe: Selenium in blood; BCd: Cadmium in blood; BPb: Lead in blood; DOC: Deoxycorticosterone; K: Potassium; Na: Sodium; Cl: Chlorine; Ca: Calcium; P: Phosphorus; Mg: Magnesium; Fe: Iron; AG: Anion gap; M: mean; SD: standard deviation. Toxic metals in blood and serum DOC and ions were logarithmically converted.


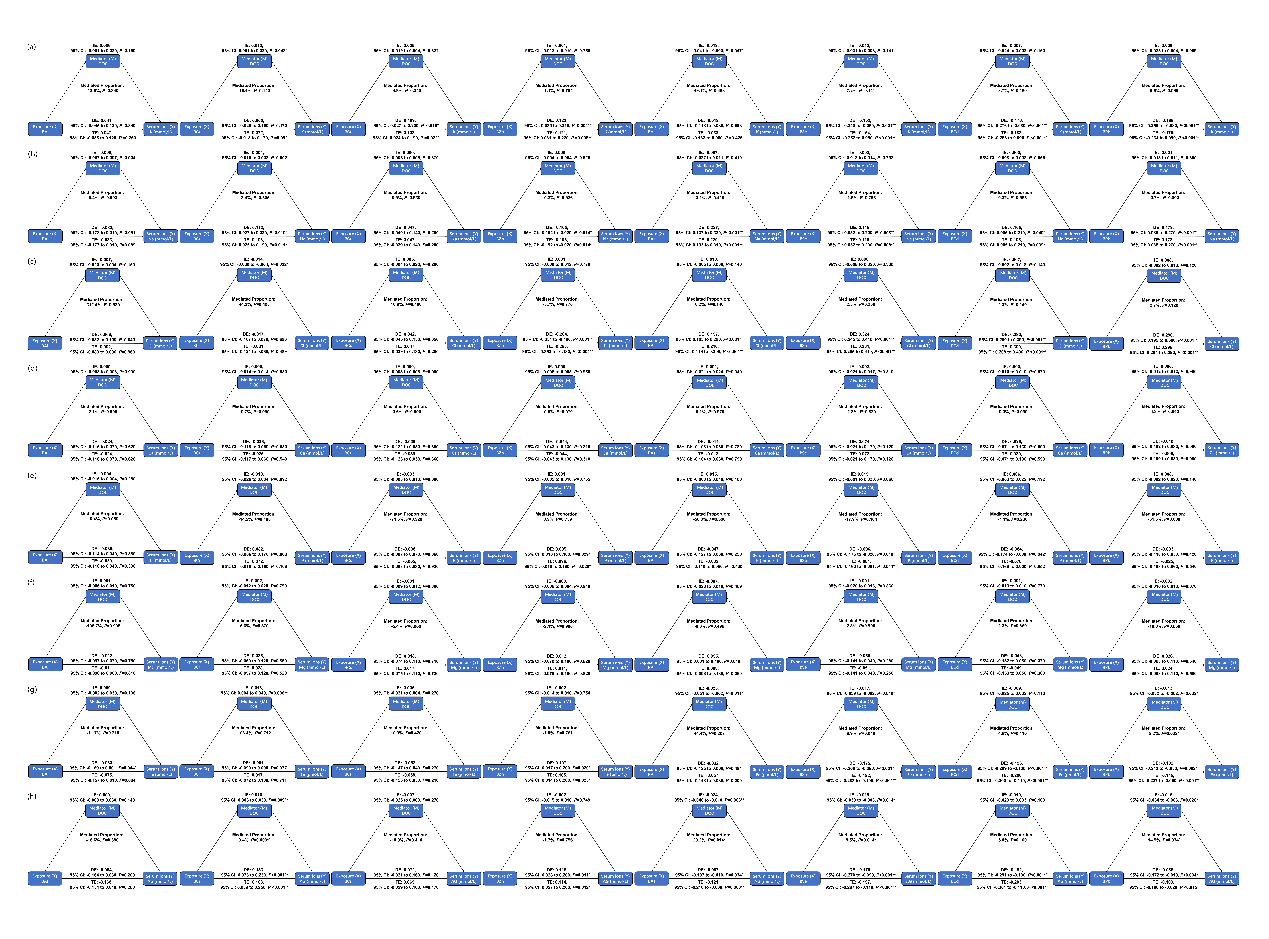


Fig. S8. Mediation effect of DOC on associations of single metal levels with serum ions. Model was adjusted for age, gender, BMI, waist circumference, cigarette smoking, and alcohol consumption. BAl: Aluminum in blood; BCr: Chromium in blood; BCu: Cuprum in blood; BZn: Zinc in blood; BAs: Arsenic in blood; BSe: Selenium in blood; BCd: Cadmium in blood; BPb: Lead in blood; DOC: Deoxycorticosterone; K: Potassium; Na: Sodium; Cl: Chlorine; Ca: Calcium; P: Phosphorus; Mg: Magnesium; Fe: Iron; AG: Anion gap; CI: Confidence interval. IE: Indirect effect; DE: Direct effect; TE: Total effect. Proportion of mediation = IE/TE. Toxic metals in blood and serum DOC and ions were logarithmically converted. *P < 0.05, **P < 0.01.

| **Table S1.** Linearity and precision data obtained for serum mineralocorticoid. | | | | | |  | |
| --- | --- | --- | --- | --- | --- | --- | --- |
| Compounds | Linear regression equation | Linear correlation coefficient r | Linear range (ng/mL) | LOD (ng/mL) | LOQ (ng/mL) | Recovery rate (%) | RSD (%) |
| BAl | y = 0.3744x-0.0214 | 0.9999 | 0–200 | 0.50 | 0.15 | 96.5–99.4 | 0.37–3.20 |
| BCr | y = 0.8328x+0.1070 | 0.9999 | 0–200 | 0.30 | 1.00 | 99.1–105.3 | 1.34–5.32 |
| BCu | y = 4.438x+0.3297 | 0.9999 | 0–2000 | 1.00 | 3.00 | 100.1–110.2 | 1.89–4.23 |
| BZn | y = 2.4381x+0.4337 | 0.9999 | 0–10000 | 5.00 | 15.00 | 94.6–103.8 | 3.08–6.43 |
| BAs | y = 0.0322x-0.0033 | 0.9999 | 0–100 | 0.50 | 1.50 | 98.8–102.6 | 1.33–5.29 |
| BSe | y = 0.8381x+0.0097 | 0.9999 | 0–200 | 0.50 | 1.50 | 98.2–106.4 | 1.01–7.32 |
| BCd | y = 0.0586x-0.0004 | 0.9999 | 0–100 | 0.08 | 0.30 | 98.3–101.5 | 1.30–3.16 |
| BPb | y = 0.2671x+0.027 | 0.9999 | 0–100 | 0.11 | 0.30 | 95.8–96.4 | 1.53–2.03 |
| DOC | y = 8370.94x-3670.71 | 0.9999 | 0–200 | 0.01 | 0.03 | 95.9–104.4 | 2.10–4.60 |
| BAl: Aluminum in blood; BCr: Chromium in blood; BCu: Cuprum in blood; BZn: Zinc in blood; BAs: Arsenic in blood; BSe: Selenium in blood; BCd: Cadmium in blood; BPb: Lead in blood; DOC: Desoxycorticosterone; LOD, Limit of detection; LOQ, Limit of quantitation; RSD, relative standard deviation. | | | | | | | |

| **Table S2.** Gradient elution procedures. | | |
| --- | --- | --- |
| Time (min) | B (%) | A (%) |
| 0.0 | 70 | 30 |
| 0.1 | 70 | 30 |
| 1.0 | 70 | 30 |
| 6.0 | 5 | 95 |
| 6.1 | 70 | 30 |
| 8.0 | 70 | 30 |
| Note: Mobile phase A: 0.1% formic acid; Mobile phase B: acetonitrile. | | |

| **Table S3.** The MRM acquisition parameters of serum mineralocorticoid. | | | | | | |
| --- | --- | --- | --- | --- | --- | --- |
| Compounds | Precursor ion (m/z) | Product ion (m/z) | DP (Volts) | CE (Volts) | ESI mode | RT (min) |
| DOC | 331.5 | 97.1*,109.2 | 174 | 27 | ESI+ | 4.12 |
| DOC, Desoxycortone; DP, Declustering Potential; CE, Collision Energies; ESI, Electrospray ionization, RT, retention time; * is quantitative ion. | | | | | | |

| **Table S4.** Normality test of toxic metals, mineralocorticoid and serum ions. | | | | |
| --- | --- | --- | --- | --- |
| Variable | Kolmogorov-Smirnova | | Shapiro-Wilk | |
|  | Z | *P* Value | W | *P* Value |
| BAl | 0.298 | < 0.001** | 0.524 | < 0.001** |
| BCr | 0.182 | < 0.001** | 0.903 | < 0.001** |
| BCu | 0.097 | < 0.001** | 0.937 | < 0.001** |
| BZn | 0.034 | 0.200 | 0.991 | 0.006** |
| BAs | 0.088 | < 0.001** | 0.879 | < 0.001** |
| BSe | 0.136 | < 0.001** | 0.828 | < 0.001** |
| BCd | 0.244 | < 0.001** | 0.721 | < 0.001** |
| BPb | 0.160 | < 0.001** | 0.687 | < 0.001** |
| DOC | 0.320 | < 0.001** | 0.382 | < 0.001** |
| K | 0.479 | < 0.001** | 0.083 | < 0.001** |
| Na | 0.324 | < 0.001** | 0.231 | < 0.001** |
| Cl | 0.309 | < 0.001** | 0.269 | < 0.001** |
| Ca | 0.182 | < 0.001** | 0.564 | < 0.001** |
| P | 0.405 | < 0.001** | 0.072 | < 0.001** |
| Mg | 0.496 | < 0.001** | 0.077 | < 0.001** |
| Fe | 0.056 | < 0.001** | 0.985 | < 0.001** |
| AG | 0.130 | < 0.001** | 0.854 | < 0.001** |
| BAl: Aluminum in blood; BCr: Chromium in blood; BCu: Cuprum in blood; BZn: Zinc in blood; BAs: Arsenic in blood; BSe: Selenium in blood; BCd: Cadmium in blood; BPb: Lead in blood; DOC: Desoxycortone; K: Potassium; Na: Sodium; Cl: Chlorine; Ca: Calcium; P: Phosphorus; Mg: Magnesium; Fe: Iron; AG: Anion gap.  The Kolmogorov-Smirnova test and the Shapiro-Wilk test were used to verify that the data is normally distributed.  **P < 0.01; *P < 0.05. | | | | |

| **Table S5.** PIP values of each metal in the BKMR model. | | | | | | | | |
| --- | --- | --- | --- | --- | --- | --- | --- | --- |
| Metals | PIP | | | | | | | |
|  | K | Na | Cl | Ca | P | Mg | Fe | AG |
| BAl | 1.0000 | 1.0000 | 1.0000 | 0.2651 | 1.0000 | 0.1008 | 0.4800 | 0.7272 |
| BCr | 0.9498 | 1.0000 | 1.0000 | 0.2419 | 0.9480 | 0.1258 | 0.4476 | 0.9992 |
| BCu | 0.9938 | 0.9528 | 0.9714 | 0.2920 | 0.9992 | 0.1928 | 0.9766 | 0.7290 |
| BZn | 1.0000 | 1.0000 | 0.9920 | 0.3033 | 1.0000 | 0.0962 | 0.9406 | 0.6258 |
| BAs | 1.0000 | 1.0000 | 1.0000 | 0.3367 | 0.8488 | 0.3274 | 0.4460 | 1.0000 |
| BSe | 1.0000 | 1.0000 | 1.0000 | 0.4673 | 0.9398 | 0.1656 | 0.9544 | 0.9300 |
| BCd | 0.9924 | 1.0000 | 1.0000 | 0.4156 | 0.9380 | 0.1634 | 0.8780 | 0.9154 |
| BPb | 1.0000 | 0.9224 | 1.0000 | 0.2911 | 1.0000 | 0.1306 | 0.7126 | 0.8250 |
| PIP, posterior inclusion probability; BAl: Aluminum in blood; BCr: Chromium in blood; BCu: Cuprum in blood; BZn: Zinc in blood; BAs: Arsenic in blood; BSe: Selenium in blood; BCd: Cadmium in blood; BPb: Lead in blood; K: Kalium; Na: Natrium; Cl: Chlorine; Ca: Calcium; P: Phosphorus; Mg: Magnesium; Fe: Ferrum; AG: Anion gap.  Toxic metals in blood and serum ions were logarithmically converted.  The model of BKMR adjusted for age, gender, BMI, waist circumference, cigarette smoking and alcohol consumption. | | | | | | | | |

| **Table S6.** PIP values of each metal in the BKMR model. | |
| --- | --- |
| Metals | PIP |
|  | DOC |
| BAl | 0.486 |
| BCr | 0.999 |
| BCu | 0.179 |
| BZn | 0.386 |
| BAs | 0.974 |
| BSe | 0.477 |
| BCd | 0.264 |
| BPb | 0.913 |
| PIP, posterior inclusion probability; DOC: Desoxycortone.  Toxic metals in blood and representative mineralocorticoid were logarithmically converted.  The model of BKMR adjusted for age, gender, BMI, waist circumference, cigarette smoking and alcohol consumption. | |
